# Supplementary figures and images for: Disruptions in gene interaction networks abolish host susceptibility to Trichostrongylus colubriformis infections in sheep
Source: PLoS Negl Trop Dis. 2025 Aug 14;19(8):e0013399. doi: 10.1371/journal.pntd.0013399 (PMC12370191; doi:10.1371/journal.pntd.0013399)

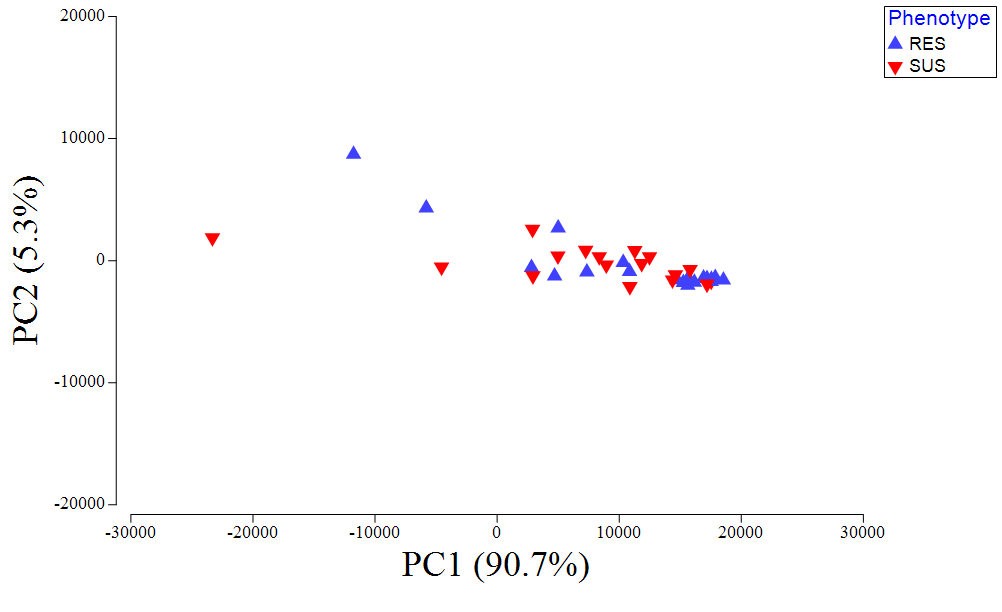

Supplement: S1 Fig — The plot was generated using top 15,000 genes sorted by their median values in transcript abundance. RES: Resistant lambs; SUS: Susceptible lambs. N = 20 per group. (TIF) [file pntd.0013399.s001.tif]

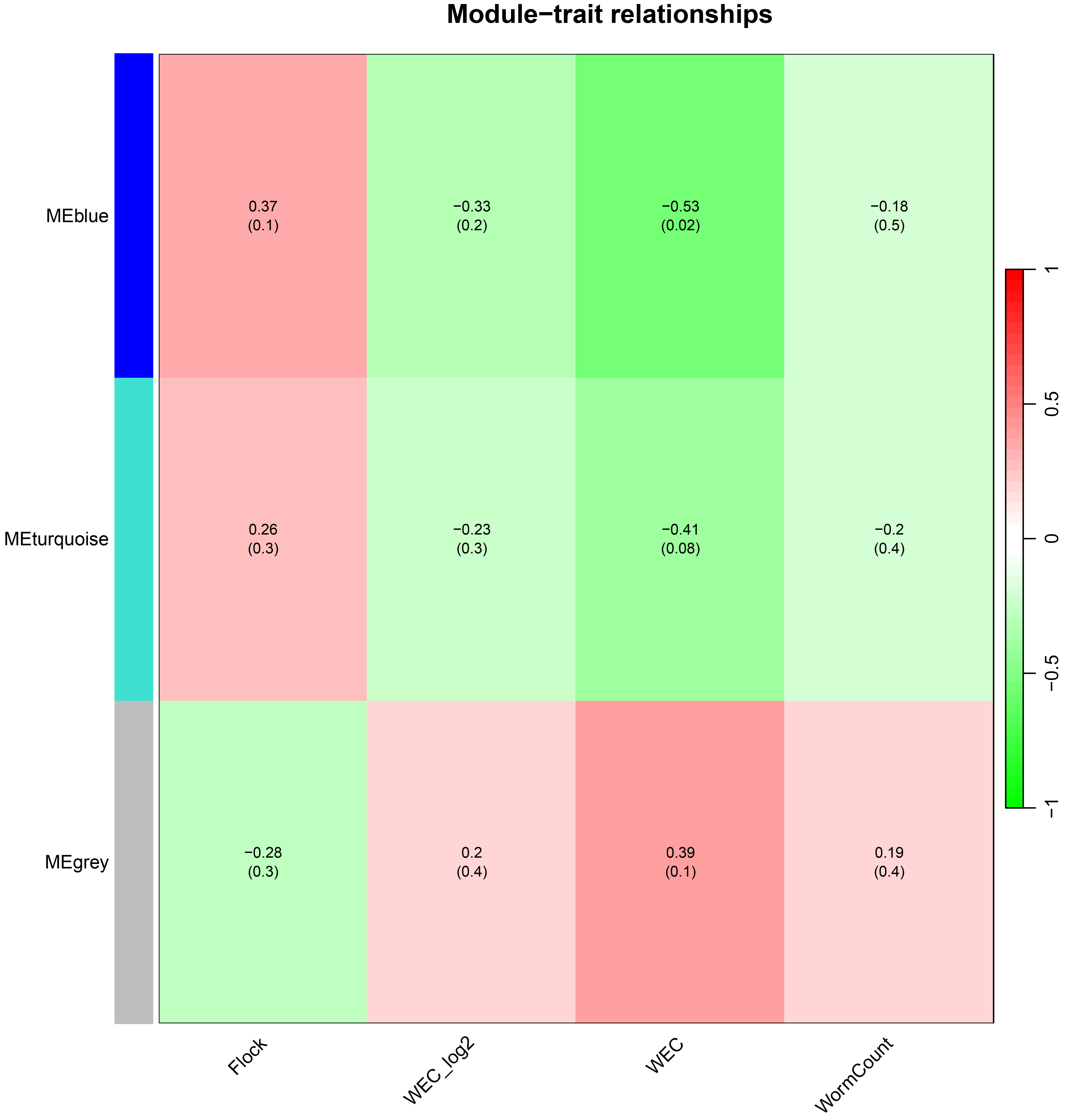

Supplement: S2 Fig — Left panel: Modules represented colors. Right: Correlation coefficient calculated using the biweight mid-correlation (bicor) method. The numbers in each cell represent correlation coefficient and P value (in the parenthesis). The blue module (MEblue) had a negative correlation with fecal worm egg counts (WEC) R = -0.53 (P = 0.02). N = 20 per group. (TIF) [file pntd.0013399.s002.tif]

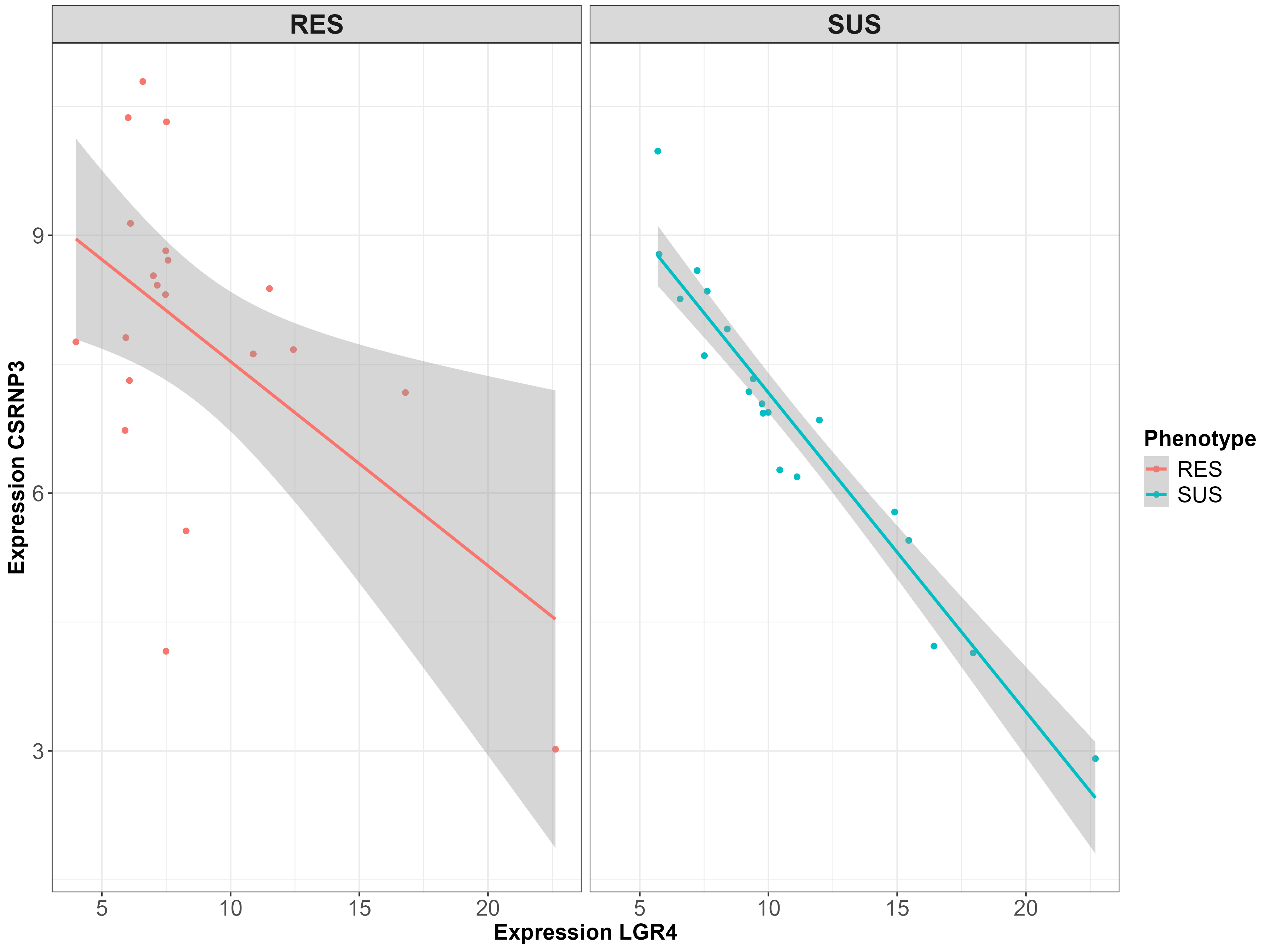

Supplement: S3 Fig — Leucine rich repeat containing G protein-coupled receptor 4 (LGR4); cysteine-serine-rich nuclear protein 3 (CSRNP3). RES: Resistant lambs. SUS: Susceptible lambs. N = 20 per group. (TIF) [file pntd.0013399.s003.tif]
