# Supplementary material for: Disruptions in gene interaction networks abolish host susceptibility to Trichostrongylus colubriformis infections in sheep
Source: PLoS Negl Trop Dis. 2025 Aug 14;19(8):e0013399. doi: 10.1371/journal.pntd.0013399 (PMC12370191; doi:10.1371/journal.pntd.0013399)
Supplement: Attachment — Submitted filename: 20250709_Responses to Reviewers Comments PLOS NTD.pdf [file pntd.0013399.s017.pdf]

Responses to Reviewers' Comments

Reviewer #1:

Introduction:
Please consider adding a brief taxonomic description of T. colubriformis (e.g., family Trichostrongylidae, morphology, site of infection). Also, the zoonotic relevance, mentioned in the author summary, could be more clearly emphasized in the main text. In support of this, we suggest including a few additional references specific to Trichostrongylus biology and pathogenesis (e.g., larval development stages, immune evasion mechanisms, host pathology).
Response: The authors thank the reviewer for his or her thorough reading of our manuscript and constructive comments. A paragraph on the zoonotic relevance of *Trichostrongylus* in humans has been added to the Introduction section (L81 to L92).

Line 81: When referring to the zoonotic potential of T. colubriformis and T. axei, consider citing human case reports or prevalence data, especially from tropical regions.

Response: Several paragraphs have been added to the Introductions section regarding human Trichostrongylosis (new L81 to L118).

Parasitological Methodology:
Some moderate adjustments are recommended for clarity:
- How were worms counted? Was the entire small intestine examined, or was a subsampling protocol used?
- Were sex and/or developmental stages of the worms recorded? This could offer insights into fecundity or developmental arrest.
Response: We appreciate the reviewer's concern. We have had a standard operating procedure (SOP) for worm counting in ruminants in place in our labs for many years.

The following section was provided in the Method section in the original manuscript:

"Entire jejunum contents and mucosal scrapings were then collected. The intestinal contents and scrapings were combined and brought to 500 ml total volume using running water. The jejunum samples were mixed well; and two 30-ml subsamples were taken, frozen at -20ºC, and later thawed and worms were counted under a dissection microscope. Total worms, including males, females, and immature larvae, were recorded".

At necropsy, the predilection site of *T. colubriformis* worms at 14 days post-infection is the jejunum. As a result, the entire length of the jejunum and the mucosal scrapings were collected. After bringing the total volume of combined jejunum contents and scrapings to 500 ml, the entire volume was mixed well after thawing. Two 30-ml subsamples were taken and counted under a dissection microscope. The sex and development stages of worms

were recorded. Because the numbers of worms from two 30-ml subsamples were very close, we believe that our method to count *T. colubriformis* worms is adequate.

Fecal Egg Count (FEC):
Please clarify the following:
- Were all animals at 0 EPG prior to infection?
- Was there any egg shedding observed post-infection in the resistant group?
- If possible, more explicitly correlate FEC data with worm counts in the Results section.

Response: The statement that "No lambs with WEC values greater than zero were observed" was provided in the original text (see new L173 to L174). As a result, no EPG were detected in all 40 lambs prior to the challenge infection. Because the lambs were sacrificed at 14 days post-infection; and before the worms become sexually mature, no egg shedding was observed in all lambs, regardless of their resistance status. The correlation between WEC and worm counts was discussed in the Discussion section.

Reviewer #2:

See Summary and General comments

Reviewer #3:

Line 136 - Haemonchus selection and Trichostrongylus selection flocks – same flock or two flocks?
Response: Two different flocks, one originally targeting *Haemonchus* while another targeting *Trichostrongylus*. Each flock has two different selection lines, resistant and susceptible. This piece of information was described in the Method section (new L162 to L163).

Line 157 – why was a sub sample of 30ml taken? Would this have captured the number of worms appropriately particularly after freezing the sample?
Response: Yes. We believe so. After thawing, the entire 500-ml samples were mixed well; and two 30-ml subsamples were immediately taken for counting. Our worm counting protocol or standard operating procedure (SOP) has been tested multiple times and perfected in detail in the past ten years.
The number of worms in each of the 30-ml subsamples was close, suggesting that these two 30-ml subsamples, or 12% of the total 500-ml samples, can faithfully represent the entire contents. As a result, counting the entire 500-ml samples is seemingly unnecessary and labor-intensive.

Reviewer #4:
(No Response)

Results

-Does the analysis presented match the analysis plan?
-Are the results clearly and completely presented?
-Are the figures (Tables, Images) of sufficient quality for clarity?

<u>Reviewer #1</u>:
- Host–Parasite Interaction:
A brief discussion linking the identified immune pathways with known mechanisms of parasite immune evasion or modulation by T. colubriformis would strengthen the parasitological interpretation of the findings.
Response: The immune pathways involved in the parasite immune evasion has been added.

Specific Points in the Text:
- Line 215: There appears to be a repetition: "resistant lambs lambs differed markedly…".
Suggested rephrasing:
"Worm establishment was significantly impaired in resistant lambs."
Response: Changed as suggested.

Figures:
- The current figures are clear. However, we suggest including images of the parasite (if available), to visually reinforce the target organism and improve accessibility for readers less familiar with T. colubriformis.
Response: We appreciate the reviewer's suggestions. We did not save any images of *T. colubriformis* worms in this particular study.

<u>Reviewer #2</u>:
See Summary and General comments

<u>Reviewer #3</u>:
Overall the result are difficult to follow. Several of the paragraphs encompassing whole pages need breaking down into smaller paragraphs with distinct thoughts/ideas. Equally including long lists of genes etc is sometimes difficult to read. The rationale/context of the results was sometimes unclear for someone with not a high level of experience in bioinformatics - some of this was better explained in the discussion, which could be moved to the results section.
Response: We thank the reviewers for pointing this out. We have made several changes and revisions to the Results section by breaking down long paragraphs. Other efforts have also been made to improve overall readability during the revision.

Line 215 – this section should start with the parasite data before discussing the transcriptome results.

Response: Revised according to the reviewer's suggestions (new L247 to L251).

Line 223 – only 7 DEG in resistant lambs – is this reflective of the sample location?
Response: Indeed, there are only 7 DEGs meeting our stringent cutoff threshold, i.e., FDR adjusted *P* value < 0.05 and the absolute fold change > 1.50, in resistant lambs. The authors do not think the limited number of DEGs detected was due to sample location, but reflective of underlying biology because all samples were collected from the same location (the jejunum) at the same time.

Line 260 – needs an explanation as to why gene interaction networks used? Also while I appreciate it is standard practice to refer t the modules as colours but is there another method that can be used that is more informative/easier to read.
Response: The authors appreciate the reviewer's insightful suggestions. We added the rationale for gene interaction networks (new L303 to L312) during the revision. There are multiple network inference methods available. In addition to WGCNA, in which the colors are used to represent modules, we also applied DGCA for network construction and analysis. We provided evidence that both methods complement each other.

Reviewer #4:
(No Response)

Conclusions

-Are the conclusions supported by the data presented?
-Are the limitations of analysis clearly described?
-Do the authors discuss how these data can be helpful to advance our understanding of the topic under study?
-Is public health relevance addressed?

Reviewer #1:
The mention of feed/phytochemical strategies to modulate gene networks is innovative and promising. Consider citing examples of plant-based anthelmintics already used in ruminants (e.g., tannin-rich forages like Lespedeza) to contextualize this idea.
Response: Several studies on tannin-rich forage plants and their antiparasitic activities have been cited during the revision.

- Lines 604–610: A table or figure summarizing key genes and their associated phenotypes (e.g., DEGs correlated with worm burden) would enhance readability for parasitologists.
Response: The information on phytochemicals as anthelmintics has been added during the revision. The genes correlated with parasitological phenotypes are provided in the supplementary tables (new Tables S9 and S10). These tables are rather lengthy and will be unlikely reader-friendly if they were inserted into the main text.

Reviewer #2:
See Summary and General comments

Reviewer #3: (No Response)

Reviewer #4: (No Response)

Editorial and Data Presentation Modifications?

Use this section for editorial suggestions as well as relatively minor modifications of existing data that would enhance clarity. If the only modifications needed are minor and/or editorial, you may wish to recommend “Minor Revision” or “Accept”.

Reviewer #1: (No Response)
Reviewer #2: See Summary and General comments
Reviewer #3: (No Response)
Reviewer #4: (No Response)

Summary and General Comments

Use this section to provide overall comments, discuss strengths/weaknesses of the study, novelty, significance, general execution and scholarship. You may also include additional comments for the author, including concerns about dual publication, research ethics, or publication ethics. If requesting major revision, please articulate the new experiments that are needed.

Reviewer #1:
The manuscript “Disruptions in gene interaction networks abolish host susceptibility to Trichostrongylus colubriformis infections in sheep” is comprehensive, well-structured, and presents a high-quality investigation into the molecular basis of resistance to this nematode in sheep. The experimental design is sound, the sample size appropriate, and the analyses appear thoroughly applied and well interpreted. The study is a valuable contribution to the field of parasitology, particularly in host–parasite interaction research. The manuscript clearly demonstrates phenotype-based differences in helminth burden between resistant and susceptible animals. The integration of parasitological data with transcriptomic analysis is commendable, especially the strong reduction in worm burden (98.8%), which supports the study's conclusions. From a practical standpoint, the findings offer potential genetic markers for selective breeding and lay the groundwork for future anti-parasitic strategies.

Response: We are grateful to the reviewer for his constructive comments and insightful suggestions. These positive and uplifting words will definitely inspire us to continue our efforts to understand host-parasite interactions utilizing these unique resource sheep populations.

Reviewer #2:
This large transcriptional dataset has an interesting experimental design and represents valuable host-response data to infection. Having 20 replicates enabled the correlation analysis, which wouldn't be possible with a smaller sample size spanning a smaller range of worm burden values. However, several improvements to the analysis are necessary to improve clarity and transparency of the data:

- Importantly, a thorough overview of the dataset should be provided. This should include a supplementary table that includes, for each sample, worm counts, egg counts and any other relevant metadata, the total number of reads sequenced, the total number of reads mapped, and the matching accessions on the SRA upload, such that the analysis could be repeated by other researchers if they wanted to. The distribution of read counts is important for interpreting the data, since samples with very low coverage will result in more genes being below detection level, and subsequently they will lack the ability to have correlation with other genes. Hopefully there is no substantial difference in these metrics between RES and SUS.

Response: We are grateful to the reviewer for constructive comments and suggestions. The metadata for each of the 40 samples sequenced has been added to Supplementary materials (new Table S1). This, in combination with the basic statistics of raw sequences for each sample (new Table S3), should paint an overall picture about the experimental design and study.

- At minimum, a PCA plot using gene expression across all genes would also show how well the samples correlate with each other, and how much the RES and SUS separate. Additionally, for example, the first principal component could be plotted against total worm counts, to show whether the overall gene expression pattern correlates with infection burden.
Response: The PCA plots using top 15,000 genes based on their median values have been generated and provided during the revision (Fig. S1). The manuscript used two different correlation methods, biweight midcorrelation (bicor) as implemented in WGCNA and Spearman rank in DCGA, to link the modules or individual genes to parasitological traits including worm burden. The module-trait correlations detected using WGCNA were indeed based on the 1st principal component to correlate modules to worm counts.

The PCA is important because of the correlation analysis. It's stated that "DGCA detected approximately four times more unique gene correlation pairs in SUS than in RES lambs." This implies that RES lambs have more noisy overall expression patterns, which should show us as them being scattered on the clustering.

Response: The reviewer raised an interesting point view that is worthy of further experimental testing. We are also somewhat perplexed by the observation that SUS lambs are more closely correlated than RES animals. We plan to conduct subsequent studies to look into this phenomenon.

Another reason that SUS may have more correlated gene pairs is that the SUS animals are undergoing substantially more of an immune response, so many sets of genes are being concurrently upregulated across animals. If the RES lambs are not responding as strongly, then they would be closer to their usual baseline uninfected transcriptional phenotype.
Response: We agree. This hypothesis warrants further research, particularly using RNAi or CRISPR to modify some key or hub genes in the interaction networks to understand causal relations.

- It would also be helpful to have a large Excel supplementary table that includes at least the read counts and FPKM values for each gene in each sample, so that readers wouldn't need to reprocess the raw data to check the expression level across samples for a gene of interest.
Response: The raw count and FPKM tables for all genes are freely accessible from the Digital Commons Data, doi: 10.17632/djjkp2743h.1. or https://data.mendeley.com/datasets/djjkp2743h/1

- Authors may consider using Ensembl biomart to bulk-download functional annotations for their genes from a variety of functional annotation algorithms such as Interpro and panther, which may provide annotations for many genes that currently lack it in the supp tables.

Response: The reviewer is correct. We did download the annotation files from the Ensembl website using Biomart (https://useast.ensembl.org/index.html).
We also conducted functional analysis including Interpro using DAVID. Only nine Interpro terms, such as ADF-H/Gelsolin-like_dom_sf (IPR029006) and Actin/actin-like_CS (IPR020902), were significantly enriched in our dataset (FDR < 0.05). Because of space limitation, we did not include the findings from Interpro in the discussion. We also performed the manual annotation of select genes. In the follow-up experiment, we will attempt to use Panther.

- Some or most of the first paragraph and a half of the discussion (up to line 485) would be more appropriate to be in the introduction.
Response: The first paragraph of the Discussion section has been removed as suggested.

- Line 536, batch effects can be considered in DESeq by incorporating them as covariates in the model. False positives and negatives are likely to be just as bad or worse in a correlation analysis, and "subtle" but significant changes can be captured easily by DESeq simply by omitting a fold change cutoff and only considering the P value, which is acceptable practice. The main reason to use correlation instead of DESeq for your analysis is that you want to look at gene changes over a continuous variable and not perform a

simple pairwise comparison. That's an acceptable reason that's more appropriate to mention than the ones in this sentence.
For example, "ME lightcran1 consisted of 96 highly interacted or correlated genes; but only one of them was differentially expressed; as a result, conventional DEG analyses alone would have missed the majority of these genes." How many of these genes had significant FDR values but just lacked the sufficient fold change cutoff?
Response: The batch effects should be minimal because all 40 samples were processed and sequenced simultaneously as a single batch in our study.

The reviewer is correct in suggesting the consideration of only unadjusted *P* values. In the preliminary analysis, we did apply three different DEG detection methods, DEseq, EdgeR, and LIMMA, with or without controlling false discovery rate (FDR). We ended up with 1,748 to 2,906 genes with raw *P* value < 0.05, too many to be discussed in detail. Our original research objective was to have a robust list of genes that can be readily validated in a separate population using qRT PCR. As a result, controlling false discovery rates is seemingly important in our study.

To answer the reviewer's question regarding "How many of these genes had significant FDR values but just lacked the sufficient fold change cutoff", we carefully examined our data and found out that none of the genes had significant FDR but did not have sufficient fold changes. There are two genes with sufficient fold change cutoff but failed to have significant FDR values (their raw *P* values < 0.05, though). Please note that there exist considerable variations in the expression values (relative abundance) among these samples, which complicated the data analysis.

- The Figure 1 caption should indicate the statistical test used (I'm assuming it's the Wilcoxon rank sum test based on the methods). Was a test for normality ran for the control group for each data type to test whether the data follows a normal distribution? If it does, then a T-test is more appropriate and will likely yield better P values.
Response: The reviewer's concern is understandable. The distribution of WEC or fecal egg counts is not normal but overdispersal in our data as well as in many published datasets.

- Figure 5 caption says that it shows GO terms with GOC in RES but not SUS, but line 358 says that it's showing ones with similar patterns between RES and SUS, which matches what the bar graphs are showing. So, it's unclear what's shown here, and the odds ratio doesn't indicate significance, which would be the more useful metric (maybe the -Log of the P value). It would be interesting to split this into the top 10 shared terms, the top 10 RES-only terms and the top 10 SUS terms rather than the top 30 shared.
- Figure 5 caption should indicate which color goes with which GO parent term.
Response: Figure 5 was automatically plotted using the DGCA wrapper function to the plotrix R package without any modification. The software does not allow us to hand pick which GO terms to be plotted; and only those over the pre-defined FDR threshold can be plotted. Odds ratios for the GO enrichment of terms were compared between these two

groups, and *p*-values were adjusted using the Benjamini-Hochberg method for each GO term category to yield false discovery rates (FDR).

The caption has been revised to include RES GOC and SUS GOC as well as the color scheme. The three colors denote one of the three GO categories, Biological Process, Cellular Components, and Molecular Function. This piece of information has been added to the figure legends.

- Figure 6 isn't useful without short GO term descriptions to understand what the data is showing, since readers can't be expected to know or manually search each GO ID. The column headers could just have the number ("003" instead of "ME003") and could have only one decimal place in the table, to make them more narrow.

Response: Figure 6 is revised to include the full names of GO terms as suggested.

- Some of the correlation network analysis is confusing. For example, Figure 7 contains the most enriched gene pairs with GoC in RES, but if you look at the hub gene RAB32, almost all of its edges are blue, which the legend indicates means there is a positive correlation in SUS but not RES. It's difficult to see in the other two, but TNFRSF1A also appears to have mostly blue edges, and OR2AT4 maybe green which means negative in SUS and nothing in RES. Overall it's not obvious from the figure how this network is enriched for RES over SUS, where we'd expect to see more purple and orange and less blue and green.

However, I understand that there is more overall correlation in SUS compared to RES, so most networks would look like this. It does look like some genes, most notably KANK1, SIPA1L1, have RES-specific edges. Because of this, it would make more sense to focus on the nodes like this with the most RES-specific edges, and perhaps have a second panel to Figure 7 which only shows the edges that are RES-specific or opposite direction between RES and SENS, to highlight the informative parts of this network.
Response: We appreciate the reviewer's concern. The genes in this module were identified by the DGCA algorithms (the function MEGENA); and the resultant Figure 7 was plotted using Cytoscape algorithm. Among all possible connections (edges) in this module, 200 edges have gain-of-correlations (GoC) in RES, the largest category. This is likely why this module was classified as the most enriched gene pairs with GoC in RES. Per the reviewers' suggestions, we added a table showing RES-specific correlations in the supplementary materials (new Table S11).

Reviewer #3:
The manuscript by Liu and colleagues describes the genes/gene networks involved in host susceptibility to Trichostrongylus colubriformis infections in sheep. This is a valid study and the approach taken by the authors is very robust, particularly the use of sheep specifically bred for resistance/susceptibility to parasite infection. However, the manuscript in written in a format and uses terminology more suitable for a bioinformatics

journal. To be published in PLoS NTD the authors should revise the manuscript to make it clearer why the different techniques were used and what the results mean. The discussion does do this in part, and several sections could be moved around to provide better context, but the authors need to think of the readership or consider a more bioinformatics journal. As detailed above the results section includes several long paragraphs that are difficult to follow, so should be edited into smaller paragraphs.

Response: We are grateful to the reviewer for careful reading of our manuscript and suggestions. Substantial improvements have been made to make the manuscript more reader friendly. Long paragraphs have been broken apart for easy reading by non-specialist readers in tropical disease and veterinary medicine.

Some final minor points:
Abstract - should mention the sample used for transcriptomics to clarify that a GWAS study wasn't carried out.
Response: Revised as suggested.

Line 440 – Merino sheep rather than Merinoland?
Response: The breed used in this study is Merino, not Merinoland or Merinolandschaf.

Reviewer #4:
The manuscript by Liu et al., describes the research on differentially expressed genes between susceptible and resistant sheep strain to the Trichostrongylus colubriformis infection. The research topic is important and interesting, but in its current state the manuscript needs thorough revision.
Response: We appreciate the reviewer for thorough reading of our manuscript. We have carefully revised our manuscript based on the reviewers' comments and suggestions and believe that the revised version has been substantially improved.

1. The list of Supplementary materials in the manuscript is missing, so it is unclear what the authors have provided as available materials and what they have not
Response: The list of supplementary materials has been added to the end of the text (after References) during the revision.

2. There are no basic sequencing statistics, in particular, the average number of reads per library, quality, the percentage of aligned reads per genome, etc. Please provide
Response: A table containing the basic sequence statistics has been provided during the revision (new Table S3). The mean number of raw reads is 60,821,935 ± 15,225,107.28 per sample (*N*= 40) with the mean percentage of Q20 is 97.44 ± 0.27.

3. Please clearly indicate how many biological and technical replicates were used in preparing the DNA libraries, how many samples were there in total for sequencing. Did you take material from one animal for each library or pool them? This information should be in the M&M section

Response: A total of 40 biological samples were sequenced (*N* = 20 per group/phenotype). No technical replicates were used. The information was clearly stated in the method section (also in new Table S3)

4. Please provide a PCA plot to demonstrate how different your samples were and what percentage of differences (variance) main components explain.
Response: A PCA plot was added during the revision (Fig. S1, new L252 to L258). The 1st component (PC1) explained 90.7% of the total variance while the 2nd component (PC2) explained an additional 5.3% variation. The cumulated variance explained by the first two components is 96%, suggesting that the PCA plots described the overall transcriptome structure rather well.

5. Please indicate the versions of all bioinformatics packages that you used throughout the text
Response: The information has been added.

6. Lines 194-195. The authors state that they did apply filtering before the Differential Gene Correlation analysis. However, it is unclear whether the authors filtered at the very beginning before identifying DEG genes? This is important and may affect the total number of DEGs. Please state clearly.
Response: We appreciate the reviewer for the details. No filters were applied before DEG analysis; and all input FPKM values for all genes were used as input files for DESeq2, unlike in DGCA (in which top 15,000 genes based on their median FPKM were used).

Do you want your identity to be public for this peer review? For information about this choice, including consent withdrawal, please see our Privacy Policy.

Reviewer #1: **Yes:** Eduardo Lopes-Torres
Reviewer #2: No
Reviewer #3: No
Reviewer #4: No
